# Biosynthetic PCL-*graft*-Collagen Bulk Material for Tissue Engineering Applications

**DOI:** 10.3390/ma10070693

**Published:** 2017-06-23

**Authors:** Piergiorgio Gentile, Kegan McColgan-Bannon, Nicolò Ceretto Gianone, Farshid Sefat, Kenneth Dalgarno, Ana Marina Ferreira

**Affiliations:** 1 School of Mechanical and Systems Engineering, Newcastle University, Newcastle-upon-Tyne NE1 7RU, UK; K.Mccolgan-Bannon2@newcastle.ac.uk (K.M.-B.); kenny.dalgarno@newcastle.ac.uk (K.D.); 2 Department of Mechanical and Aerospace Engineering, Politecnico di Torino, 10129 Turin, Italy; nicolo.cerettogianone@studenti.polito.it; 3 Department of Medical Engineering, School of Engineering, University of Bradford, Bradford BD7 1DP, UK; F.Sefat1@bradford.ac.uk

**Keywords:** biosynthetic, collagen, poly(ε-caprolactone), conjugation, electrospinning, tissue engineering

## Abstract

Biosynthetic materials have emerged as one of the most exciting and productive fields in polymer chemistry due to their widespread adoption and potential applications in tissue engineering (TE) research. In this work, we report the synthesis of a poly(ε-caprolactone)-*graft*-collagen (PCL-*g*-Coll) copolymer. We combine its good mechanical and biodegradable PCL properties with the great biological properties of type I collagen as a functional material for TE. PCL, previously dissolved in dimethylformamide/dichloromethane mixture, and reacted with collagen using carbodiimide coupling chemistry. The synthesised material was characterised physically, chemically and biologically, using pure PCL and PCL/Coll blend samples as control. Infrared spectroscopy evidenced the presence of amide I and II peaks for the conjugated material. Similarly, XPS evidenced the presence of C–N and N–C=O bonds (8.96 ± 2.02% and 8.52 ± 0.63%; respectively) for PCL-*g*-Coll. Static contact angles showed a slight decrease in the conjugated sample. However, good biocompatibility and metabolic activity was obtained on PCL-*g*-Coll films compared to PCL and blend controls. After 3 days of culture, fibroblasts exhibited a spindle-like morphology, spreading homogeneously along the PCL-*g*-Coll film surface. We have engineered a functional biosynthetic polymer that can be processed by electrospinning.

## 1. Introduction

The field of tissue engineering (TE) is primarily concerned with the fabrication of scaffolds for cellular growth and tissue regeneration. Scaffolds provide physical support for the growth and development of new tissue, thus, the mechanical, physicochemical and biological properties are key considerations when selecting scaffold materials [1,2]. Synthetic polymers such as poly-ε-caprolactone (PCL), polylactic acid (PLA), poly lactic-*co*-glycolic acid (PLGA), etc., have been widely used in TE research as they have a variety of physicochemical and mechanical properties that allow for a range of TE applications [3,4,5,6]. While synthetic polymers possess attractive mechanical and physicochemical properties, their smooth outer surface and unnatural molecular composition impede cellular adhesion and growth [7,8]. Proteins present in the extra cellular matrix bear domains to which integrin in the cellular membrane can attach, allowing cells to proliferate.

A well-known TE approach is the manufacturing of 3D scaffolds composed of natural and synthetic polymers, in order to exploit their complementary physical–chemical properties. As an example, PCL has been extensively used in scaffolds due to its biocompatibility, bioresorbability, and good mechanical properties [9,10]; however, like many synthetic polymers, it is characterised by poor cellular adhesion [7,11]. On the other hand, as a typical example of natural polymers, collagen (Coll) is the most common protein in the extracellular matrix (ECM) and bears the RGD domain which stimulates cellular adhesion [12].

A number of different strategies to fabricate the PCL–collagen scaffold have been described. The first approach, concerning the preparation of a blend, was reported for the first time in 2002 when Dai et al. mixed collagen with PCL to manufacture tissue-engineered skin substitutes. In this work, they studied the interaction between the two polymers by varying their weight ratio (1:4, 1:8 and 1:20 w/w Coll/PCL). They found that fibroblasts attached and proliferated on all composites, reaching the optimal condition at the 1:20 blend ratio after 8 days of culture [13]. Although, in the last 15 years, several works on PCL/Coll blends for the preparation of dense films have been heavily reported in literature [14], most of the PCL and collagen combinations have been for the preparation of fibrous constructs using electrospinning. Several approaches have also been proposed for improving the miscibility of the two polymers. Dippold et al. described the formation of PCL/collagen meshes via electrospinning using environmentally benign diluted acetic acid as solvent, and using ultrasonic treatment to enhance the weak solubility of PCL in the selected solvent [15]. Lee developed a blend scaffolding system able to withstand physiologic vascular conditions consisting of high pressure and flow. The composite scaffolds, fabricated by mixing a high molecular weight PCL and type I collagen (1:1 weight ratio), possessed good biomechanical properties and demonstrated long-term stability under a continuous perfusion bioreactor system for up to 4 weeks [16]. Furthermore, the same nanofibrous membranes were demonstrated to maintain structural integrity and potency for 1 month when implanted in rabbits as an arterial bypass graft [17]. Electrospun PCL–collagen nanofibrous membranes have been used for the neural differentiation of adipose and bone marrow-derived rat mesenchymal stem cells (Ad-MSCs and BM-SCs). Collagen enhanced cell proliferation, although it did not support MSC attachment during the 4 h attachment period, and its addition to the PCL supported the improvement of the neural and glial differentiation, mainly, of induced Ad-MSCs [18]. Finally PCL/gelatin membranes have been successfully electrospun on a commercial polyurethane dressing (Tegaderm™, Bracknell, UK, 3M Medical) in order to create a nanofiber construct for growing autogenous fibroblast populations to provide a new solution for dermal wound treatment [19].

An alternative approach to the mixing is to coat PCL electrospun fibres with collagen. This effect of nanofiber surface coating has been evaluated by the research group of Prof Ramakrishna that prepared collagen-coated PCL nanofibers (in the form of a core-shell structure) by a coaxial electrospinning technique. It was found that coatings of collagen definitely favoured cell proliferation and the efficiency was dependent of the means of coating. As compared to pure PCL, the core-shell structure encouraged cell migration inside the scaffolds as observed by Scanning Electron Microscopy (SEM) [7]. A similar core-shell PCL/Coll structure has been described recently by Kim [20], combining the use of an electro-hydrodynamic jet and a bio-printing process. Briefly, they used a core/shell nozzle in the process and manipulated the various concentrations and flow rates of PCL solution in the core region so that variable mechanical properties of the scaffold could be achieved. To demonstrate its suitability as a biomedical scaffold, type I collagen was grafted chemically on the PCL scaffold surface and tested using preosteoblasts (MC3T3-E1 cells). The results demonstrated that the fibrous Coll coated scaffold provided significantly greater cellular activity. Similar PCL scaffolds, prepared by an electro-hydrodynamic jet, followed by collagen grafting mediated by polydopamine, have been reported in literature to accurately replicate the internal three-dimensional microstructure of cartilage. The surface functionalization made the PCL scaffold hydrophilic and favourable for cell adhesion. The chondrocytes maintained their healthy phenotypes within the collagen-grafted PCL scaffold only, showing an increased production of sulphated glycosaminoglycan and highly expressed type II collagen. These results demonstrated that collagen had a positive role in stimulating chondrogenesis [21].

Although blends of PCL and collagen have been used to fabricate films and scaffolds—most commonly by mixing, coating or surface functionalization—these techniques limit collagen presence to the outside of scaffolds or are susceptible to leaching. Furthermore, little research has been conducted on the conjugation of synthetic polymers (i.e., PCL, PLA, and PLGA) with natural polymers. A successful attempt to prepare a biosynthetic copolymer has been described by Wiens et al. [22]. In this work, a Chitosan-*g*-PCL biosynthetic-based material was developed to improve bone regeneration. The material was biocompatible with enhanced cell mineralization and ALP activity.

**Figure 1:**
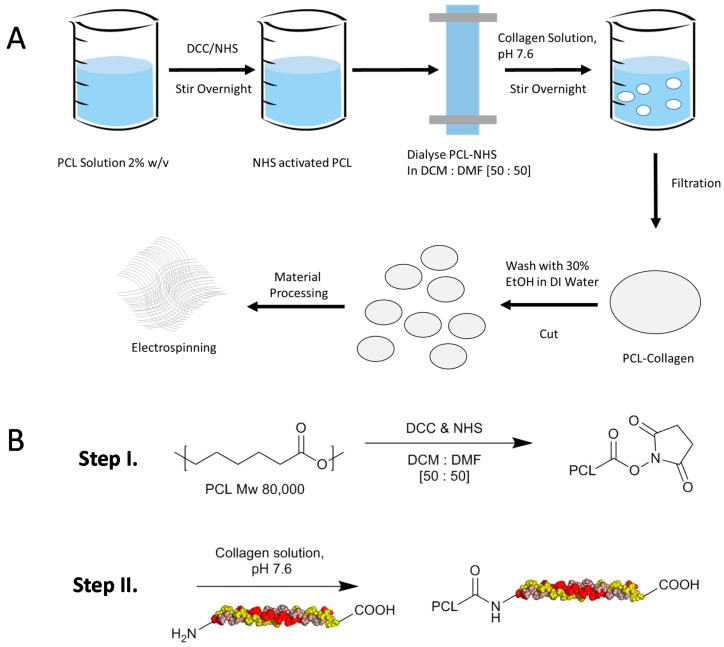
Schematic representation of the Poly-ɛ-caprolactone-*graft*-Collagen (PCL-*g*-Coll) material preparation. (**A**) Step-by-step preparation process of formulated biosynthetic material; and (**B**) Schematic representation of PCL and collagen coupling reaction by carbodiimide chemistry.

Therefore, moving beyond the state of the art, the aim of the work described in this paper was the development of a biosynthetic PCL-*g*-collagen copolymer, capable of being processed using conventional TE manufacturing techniques. To achieve this aim, carbodiimide coupling chemistry has been exploited in order to graft collagen onto PCL, as shown in Figure 1.

This approach offers a new biosynthetic material with good mechanical and biological properties due to the combination of both materials. The conjugated PCL-*g*-Coll can be processed as a unique bulk biomaterial, characterised by an even distribution of collagen throughout, in order to be used for different conventional and unconventional techniques for the manufacturing of suitable scaffolds for tissue engineering. In this paper, the synthesised material has been characterised as a cast film for the evaluation of physical–chemical (XPS, FTIR/ATR, Contact Angle, Tensile test) and biological properties (qualitative Live/Dead assay, metabolic activity and morphology by DAPI (4’,6-Diamidino-2-Phenylindole) and Phalloidin staining). Finally, preliminary tests on synthesised bio-synthetic material processing were performed by electrospinning techniques and characterised in terms of morphology and presence of collagen.

## 2. Results

### 2.1. Physico-Chemical Characterisation of Casted Films

#### 2.1.1. Fourier Transform Infrared Spectroscopy in Attenuated Total Reflection Mode (FT-IR/ATR)

**Figure 2:**
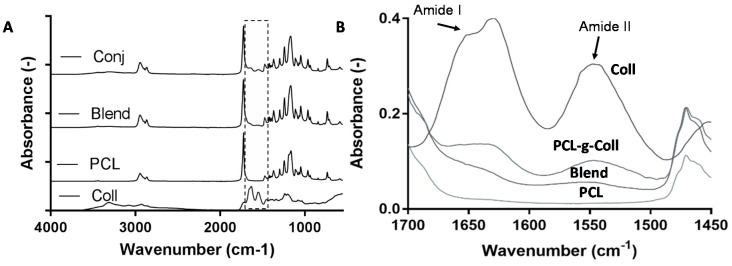
(**A**) Fourier Transform Infrared Spectroscopy in Attenuated Total Reflection Mode (FTIR/ATR) of PCL, Collagen, PCL/Coll blend, PCL-*g*-Coll samples; (**B**) The typical band of Amide I and II in order to detect the presence of collagen.

FT-IR/ATR spectra recorded on pure PCL, Coll, PCL/Coll and PCL-*g*-Coll samples are shown in Figure 2A, while a zoomed-in image of the typical band of the collagen (1700–1450 cm^−1^) is shown in Figure 2B. The spectrum of PCL showed the characteristic bands at 2960 and 2870 cm^−1^, due to the asymmetric and symmetric C–H stretching vibration. The peaks at 1724 and 1190 cm^−1^ are due, respectively, to C=O and (C=O)–O stretching vibrations. The δ CH bond vibrations were observed at 1475 cm^−1^. The collagen spectrum revealed the following bands: 1650–1665 cm^−1^ (C=O) corresponding to Amide I; 1530–1550 cm^−1^ corresponding to Amide II δ (NH); 3325–3330 cm^−1^ for Amide A (N–H); the peaks at 2956 cm^−1^ corresponding to functional group CH_3_; 2924 cm^−1^ to CH_2_; 1239 cm^−1^ to Amide III δ (NH); and 3080 cm^−1^ to Amide B (CH_2_). Those functional groups correspond to collagen [23,24].

The conjugated spectrum showed the main peak of the PCL (described above) with the appearance of the corresponding collagen peaks of Amide I and Amide II. Particularly the C=O vibrations of the conjugated samples observed at 1630 cm^−1^ presented a shift of about 20 cm^−1^ lower than collagen alone. The addition of collagen was also supported by the broadening of NH vibrations and shift of C=O vibration of PCL to a slightly lower value. On the other hand, in the blend spectrum, the intensity of Amide I and II was considerably decreased.

#### 2.1.2. X-ray Photoelectron Spectroscopy (XPS)

**Figure 3:**
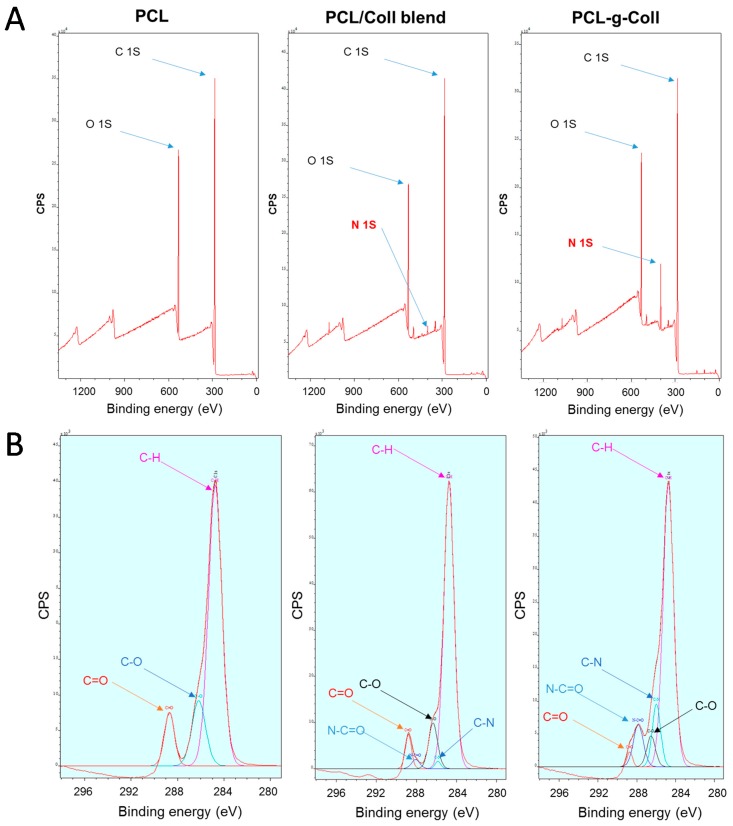
(**A**) XPS Survey; (**B**) XPS high resolution of PCL, PCL/Col blend and PCL-*g*-Coll samples.

XPS spectrum showed the characteristic elements of PCL (C1*s* peak at around 285.5 eV and O1*s* peak at 532 eV) (Figure 3A). After addition of the collagen by blending or chemical conjugation, an N1*s* peak at 399.5 eV can be observed, indicating that Coll was successfully introduced. However, a higher N content was evidenced in the conjugated sample (4.87 ± 0.65% for the PCL-*g*-Coll and 1.12 ± 0.24% for the PCL/Coll). Furthermore, Figure 3B shows the high resolution spectra for C1*s* along with the curve showing five peaks attributed to the different Carbon oxidation states: (1) 284.7–285.0 eV; (2) 286.0–286.5 eV; (3) 286.8−287.0 eV; (4) 284.1–284.5 eV and (5) 288.5–289 eV, corresponding to –C–H or –C–C– bonds, to the C–N bond, to the –C–O– bond, to –N–C=O (amide) and to C=O groups respectively [25].

The C1*s* core-level spectra of pure PCL film showed a major neutral carbon (C–H) component at a binding energy 284.6 eV and two minor components at binding energy 286.5 eV (C–O) and at binding energy 288.8 eV (C=O) respectively. For the spectra of PCL/Coll and PCL-*g*-Coll, two new peaks associated with C–N and O=C–N appeared, at a binding energy of 285.7 eV and 287.6 eV respectively. Moreover, as shown in Table 1, the content of these components varied significantly. The concentration of N–C=O and C–N increased drastically in the conjugated samples (8.52 ± 0.63% and 8.96 ± 2.02% for 3.59 ± 0.87% and 1.55 ± 0.19% respectively for the blend). Furthermore, for PCL-*g*-Coll, the component at 284.9 eV corresponding to C–C bonds decreased, reaching a final value of (71.82 ± 1.27%) and the component at 286.5 eV attributed to C–O bonds decreased, reaching a final value of (7.64 ± 0.63%), suggesting the addition of collagen.

#### 2.1.3. Contact Angle (CA) Measurement

**Figure 4:**
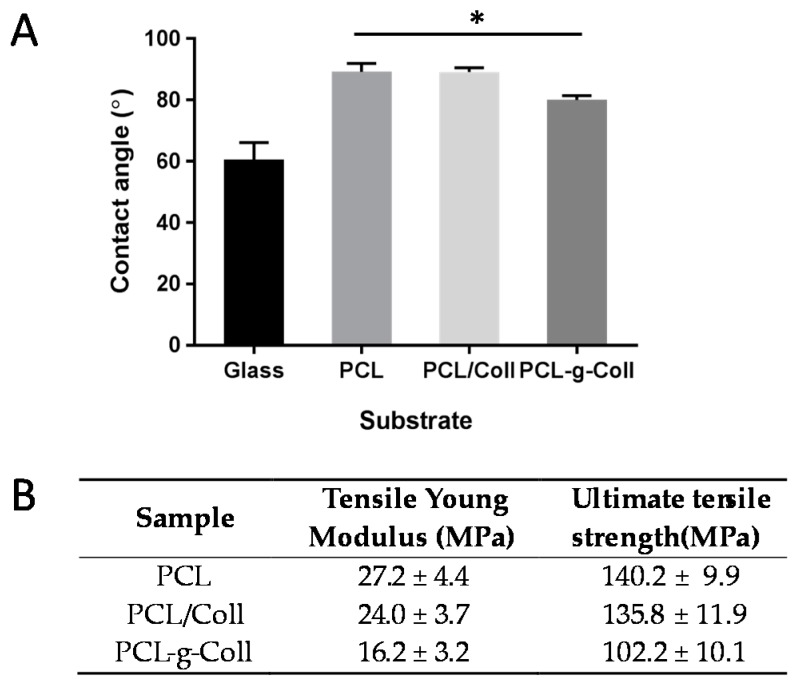
(**A**) Contact angle and (**B**) Mechanical properties (Tensile test and DMS) of PCL, PCL/Coll blend, PCL-*g*-Coll (* *p* < 0.05).

The wettability of the PCL, blend and conjugated samples was evaluated by static contact angle analysis (Figure 4A). PCL exhibited the highest contact angle 89.2 ± 2.7°, followed by PCL/Coll with a contact angle of 88.4 ± 1.4°. The synthesised PCL-*g*-Coll showed a lower CA (80.0 ± 1.4°).

#### 2.1.4. Tensile Tests

The Young’s module for the PCL, PCL/Coll and PCL-*g*-Coll was 27.2 ± 4.4 MPa, 24.0 ± 3.7 MPa and 16.2 ± 3.2 MPa, respectively (as shown in Figure 4B). This indicates little difference between the pure PCL and blend films, while the presence of collagen in PCL-*g*-Coll decreased the tensile Young’s modulus by about 40% of the PCL value. Ultimate tensile strength of PCL was measured at 140.2 ± 9.9 MPa as compared to 102.2 ± 10.1 MPa for the conjugated sample, indicating a significant drop in tensile strength due to the addition of collagen. In comparison, the blend film did not show significant differences with respect to PCL.

#### 2.1.5. Collagen Staining and In Vitro Degradation Tests

**Figure 5:**
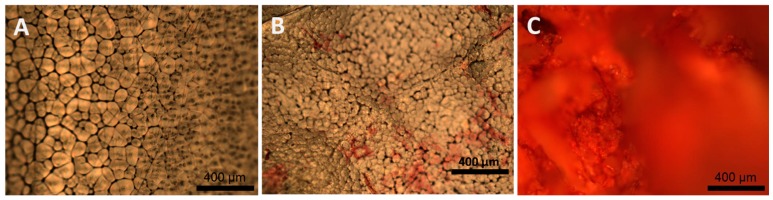
Collagen staining of (**A**) PCL; (**B**) PCL/Coll blend and (**C**) PCL-*g*-Coll with Sirius Red assay. Scale bars correspond to 400 μm.

A Sirius red assay was used to identify the presence of collagen since it recognises different types of collagen but not gelatin (denatured or unfolded collagen). The presence of red-stained collagen was evaluated in PCL (as control), PCL/Coll blend and PCL-*g*-Coll (Figure 5). A high collagen content was observed on PCL-*g*-Coll substrates (Figure 5C) stained red by Sirius red assay, whilst PCL/Coll blend evidenced a slight collagen content and PCL (control) showed no collagen content.

**Figure 6:**
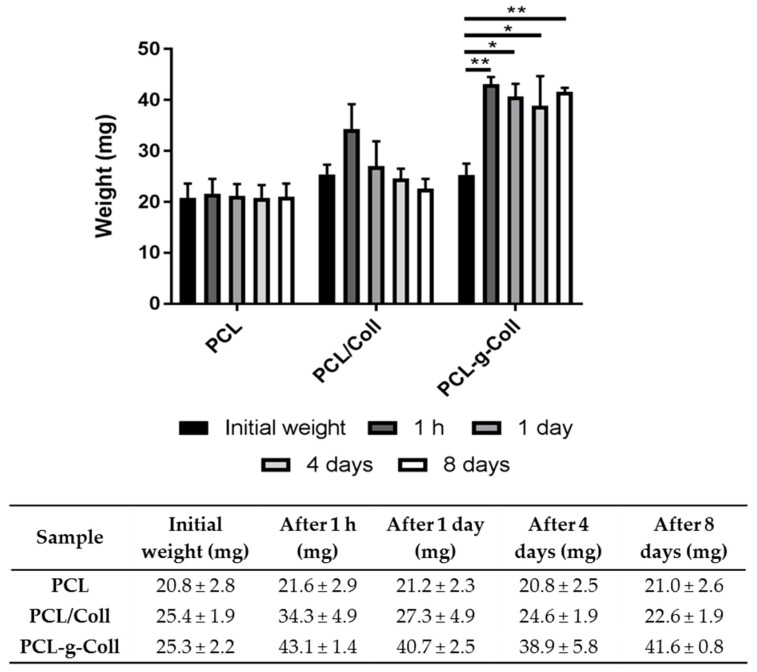
Sample weight after in vitro degradation tests in PBS at 37 °C (*n* = 3, * *p* < 0.05 and ** *p* < 0.001). The values of each type of sample are normalised against their corresponding initial weight.

The stability of the films was studied after measuring the sample weight after incubation in phosphate buffered saline solution (PBS) (pH 7.4) for fixed interval times. As shown in Figure 6, PCL material does not show significant differences in weight throughout the incubation period. However, the presence of collagen in PCL/Coll blend and PCL-*g*-Coll evidenced an increase in the initial weight after 1 h of incubation. Interestingly, PCL-*g*-Coll maintained this weight (1 h) throughout the incubation period, while PCL/Coll decreased it significantly over the following incubation days (22.6 ± 1.9 mg after 8 days), showing a similar weight of pure PCL film.

#### 2.1.6. Cell Culture and Cytoskeletal Staining

**Figure 7:**
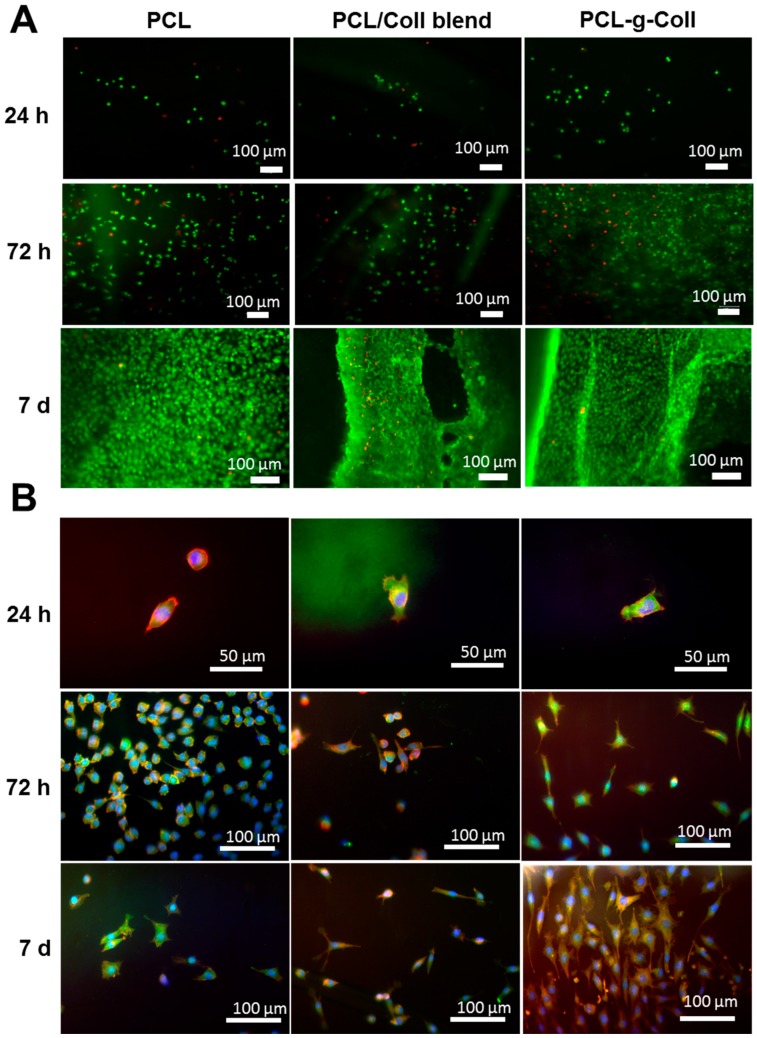
L929 cell response to PCL, PCL/Coll blend, PCL-*g*-Coll: (**A**) Live/Dead assay (scale bar = 100 μm); (**B**) Cellular staining with rhodamine phalloidin for the actin filaments (red), focal adhesion protein vinculin (green) and nuclei counterstained with DAPI (blue).

The viability of L929 fibroblast cells cultured on PCL, PCL/Coll blend and PCL-*g*-Coll conjugate was qualitatively assessed by fluorescence-based Live/Dead assay. Figure 7A shows the representative fluorescence images of live (stained green) and dead (stained red) fibroblast cells on different substrates after 24 h, 72 h and 7 days of cell culture. No major qualitative differences in cell viability were found between different substrates. The formation of a cellular monolayer was observed after 7 days of incubation on all three polymeric substrates. Fluorescent staining of L929 actin filament (red by phalloidin), nucleus (blue by DAPI (4’,6-Diamidino-2-Phenylindole)) and focal adhesion (green by vinculin) adhered to PCL, PCL/Coll blend and PCL-*g*-Coll conjugate after 24 h, 72 h and 7 days is shown in Figure 7B. The fluorescence images show more rounded cell morphology on PCL substrate after 24 h and 72 h of incubation. It can be observed that cells seeded onto PCL/Coll blend and PCL-*g*-Coll exhibited typical elongated morphology and extensive cell spreading. This morphology was also confirmed after one week of incubation. The presence of vinculin in the periphery and centre of the cell indicates a strong focal adhesion to the PCL/Coll blend and PCL-*g*-Coll surfaces.

**Figure 8:**
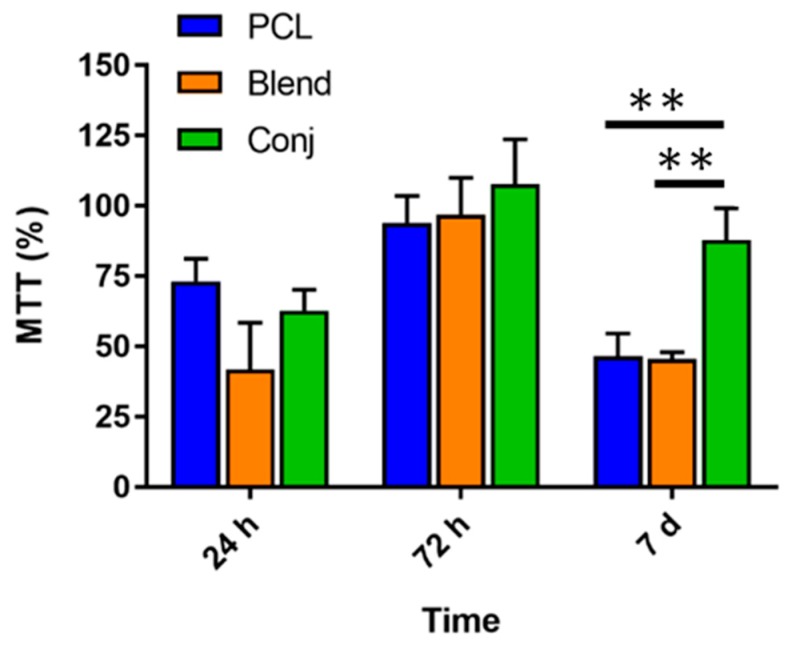
Metabolic activity of L929 cell response to PCL, PCL/Coll blend, and PCL-*g*-Coll (** *p* < 0.001) measured by MTT (3-(4,5-Dimethylthiazol-2-yl)-2,5-Diphenyltetrazolium Bromide) assay. The values are normalised against tissue culture plastic control.

The L929 cellular metabolic activity was evaluated by MTT (3-(4,5-Dimethylthiazol-2-yl)-2,5-Diphenyltetrazolium Bromide) assay after 24 h, 72 h and 7 days of cell incubation (Figure 8). Metabolic activity of cells cultured on PCL, PCL/Coll blend and PCL-*g*-Coll conjugate did not show statistically significant difference after 24 h and 72 h of incubation. However, cells cultured on PCL-*g*-Coll showed higher metabolic activity (*p* < 0.05) in comparison to PCl and PCL/Coll blend after 7 days.

### 2.2. Characterisation of PCL/Coll Electrospun Membranes

**Figure 9:**
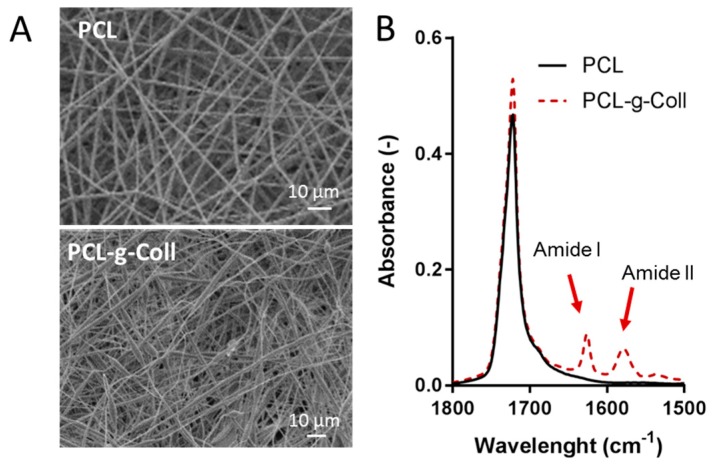
(**A**) Scanning Electron Microscopy (SEM) micrographs of the electrospun membranes composed by PCL (above) and PCL-*g*-Coll (below); (**B**) Zoomed-in image of the significant wavelength band of the FTIR/ATR for PCL and PCL-*g*-Coll electrospun membranes.

In this work, composite membranes based on PCL PCL-*g*-Coll have been obtained by electrospinning. After optimization of the process parameters (solution concentration 10%, voltage between 10–15 kV, distance 18 cm and flow rate 2.5 mL/h) smooth nano- and micro-fibres were formed for both samples (Figure 9A). Particularly the electrospun fibres showed a random morphology with a fibre diameter of 2.2 ± 1.3 μm and 1.8 ± 1.1 μm for PCL and the conjugated sample respectively. The addition of collagen was demonstrated by the obtainment of FTIR/ATR spectra on the mesh surfaces (Figure 9B), where the Amide I and II was detected in the PCL-*g*-Coll, which represented the characteristic peaks of collagen as mentioned before in section (Section 2.1.1)

## 3. Discussion

### 3.1. Evaluation of the Successful Synthesis of PCL-g-Coll

The successful collagen grafting to PCL has been assessed by XPS and FTIR-ATR. The typical bands of amide I and II of collagen were detected in both PCL-*g*-Coll and PCL/Coll blend; lower intensity was found in the latter. This result was also confirmed by XPS due to the presence of an N1*s* peak in both samples. However, a significantly higher concentration of N–C=O and C–N groups attributed to the presence of collagen amide bonds was observed in the PCL-*g*-Coll conjugated samples, in comparison to PCL and PCL/Coll blend. This work also demonstrated the advantages of using bulk bioconjugated materials rather than blends. Following several equal washes of PCL/Coll blends and PCL-*g*-Coll, it was evidenced by XPS and FTIR-ATR that the amount of collagen in the PCL/blends is reduced considerably throughout the washes while the bulk conjugated PCL-*g*-Coll remains attached to the collagen molecules. The shift down of about 20 cm^−1^ observed in PCL-*g*-Coll spectra when compared to collagen alone might be caused by a covalent interaction between the PCL and collagen molecules that weakens specific molecular interactions present in and between collagen molecules, such as hydrogen bonding and dipole–dipole interactions [26]. This type of interaction is related to the capability of the protein, when attached to the PCL material, to interact with water molecules and hydrate.

Contact angle analysis is a sensitive technique for studying the wettability of surfaces. In this work, contact angle measurements show higher and improved hydrophilic properties on PCL-*g*-Coll conjugated material than on PCL and PCL/Coll blend. Moreover, no significant differences were observed between these last two samples. These results are evidence of enhanced PCL wettability properties achieved by the successful grafting of collagen molecules.

The mechanical properties of PCL, PCL/Coll blend and PCL-*g*-Coll conjugated evaluated by tensile testing show interesting results. While no significant differences of Tensile Young’s Modulus and Ultimate Tensile Strength were detected between PCL and PCL/Coll blend, a significant decrease was observed on conjugated PCL-*g*-Coll when compared to PCL and blend. Collagen, like any natural polymer, lacks good mechanical properties when compared to synthetic ones [27,28], so decreased PCL mechanical properties are to be expected. Despite this effect, most PCL-*g*-Coll biosynthetic materials have good mechanical properties (comparable to several synthetic materials) for a wide range of tissue engineering applications [29].

In order to confirm the presence of collagen, Sirius Red staining was used. This dye binds collagen side-chain groups of basic amino acid residues and unfolded collagen (gelatin) is not recognised by this assay. Figure 5 shows the presence of collagen on PCL-*g*-Coll, and to a much lesser extent on the PCL/Coll blend. However, additional tests would be helpful to confirm the presence of the collagen triple-helix conformation.

In vitro degradation tests showed an interesting and clear effect of conjugated collagen on PCL material properties. As described in the results section, PCL maintained the material’s initial weight, demonstrating stability and low biodegradability over 8 days of incubation. However, the presence of collagen improved the capability of both materials, PCL/Coll and PCL-*g*-Coll, to interact with water by increasing the materials’ initial weight after 1 h of incubation. Interestingly, while PCL/Coll decreased this weight sharply, achieving the initial dry material weight over the following days, PCL-*g*-Coll was able to maintain it, suggesting stability and notable improvement of material interaction with water. Collagen is a biomolecule that plays a key role in the hydration and homeostasis of the ECM due to interactions with water and ions [30,31]. This effect is evident on blend and conjugated materials. However, the significant weight decrease observed on PCL/Coll material starting at day 1 when compared to 1 h incubation can be attributed to a potential collagen release, impacting the material weight and capability to absorb/interact with water. In contrast, PCL-*g*-Coll showed great stability throughout the 8 days of incubation, evidencing a capability to interact with water (by increasing the initial weight after 1 h of incubation) and maintain retained water over the following incubation days. This feature is relevant for the mechanical and biological properties and performance of the material in tissue engineering applications [32,33].

One of the purposes of this work was to evaluate how the presence of collagen could influence fibroblast cell-like behaviour. Collagen is known for its good biological properties, as a main structural protein in the ECM, and as a key protein related to cell migration [34]. In terms of cell viability, no evident differences were found between the samples—PCL, PCL/Coll blend and PCL-*g*-Coll conjugated—by the qualitative LIVE/DEAD analysis. The three materials demonstrated no cytotoxic effect throughout the incubation period; as observed in the fluorescence images, there was a great number of viable cells (green) in comparison to dead cells (red). The typical great proliferation of the L929 cells—achieving a thick monolayer of viable cells after 7 days on the different substrates—was also evident. Interestingly, greater metabolic activity was found on the conjugated PCL-*g*-Coll material, compared to the other substrates. This can be attributed to the presence of collagen, which in PCL-*g*-Coll is covalently attached to PCL, impacting the material’s biocompatibility over time.

Fluorescence images show the cells’ morphology attached to the different samples. In general, typical spindle-like-shaped L929 cells adhering to substrates were observed at 24 h and 72 h. The nucleus staining by DAPI (stained by blue) found on the different samples evidences the attachment and cell spreading over the surfaces [35]. It can be qualitatively seen from the vinculin staining (green) that the cells attached to PCL/Coll blend and PCL-*g*-Coll surfaces showed a higher concentration of vinculin at the cell periphery and centre after 24 h of incubation, in comparison to PCL. The less noticeable vinculin detected on cells suggests a lack of strong focal adhesion contacts. Furthermore, cells remained in a more spherical morphology on PCL substrates than in PCL-*g*-Coll after 72 days of incubation. These results suggest a strong focal cell contact—particularly to PCL-*g*-Coll—as a consequence of the interaction between cell surface receptors and the collagen GFOGER sequence. The results of this work on the stability of PCL-*g*-Coll material for tissue engineering applications show improved material physical, chemical and biological properties, with significant enhancement of metabolic activity and cell spreading after one week of incubation, when compared to traditional surface functionalisation techniques [36,37].

### 3.2. Preliminary Considerations on PCL-g-Coll as Base Materials for Electrospun Scaffolds Preparation

The PCL-*g*-Coll has also been considered for the preparation of highly porous membranes by electrospinning. This conventional method has been widely accepted as the simple and less expensive method to fabricate random or aligned fibrous matrices through the extrusion of the solution from a needle by a high voltage electric field [2]. By tuning electrospinning processing parameters, it is possible to modify fibres’ morphology and dimensions to enhance the spun morphology for the promotion of a positive cellular response [38]. Uniform membranes were obtained, as shown by SEM images, with fibre size comparable with pure PCL meshes. Additional tests are required to test the mechanical properties (by Dynamic Mechanical Analysis, DMA) to evaluate their stiffness and elasticity as suitable membranes in tissue engineering applications. Literature reports that electrospun meshes with a Young’s modulus of 15–20 MPa can be applied for skin tissue regeneration [39].

Finally, electrospinning suggests that a chemical bond formed between the carboxyl groups of the surface of PCL with the amino groups of the collagen may result in better collagen stability on the PCL fibre’s surface even after extensive washing in distilled water; this is promising for their PCL-*g*-Coll application in tissue engineering.

## 4. Materials and Methods

### 4.1. Materials

Poly-ε-caprolactone (PCL) (440744, 80 kDa), Collagen Type I from calf skin (C9791), Dicyclohexylcarbodiimide (DCC) (D80002), *N*-hydroxysuccinimide (NHS) (130672), *N*,*N*-dimethylformamide (DMF) (227056), Sodium Hydroxide (NaOH) (S2770), Dichloromethane (DCM) (270997), (3% v/v) acetic acid (1005706), Ethanol (652261), Phosphate Buffered Saline (PBS) (PBS1) and Thiazolyl Blue Tetrazolium Bromide (M2128) were purchased from Sigma-Aldrich (Gillingham, UK). The ultrapure water employed throughout the experiments was obtained with a Milli-Q^®^ (Watford, UK) Integral system equipped with a BioPak^®^ ultrafiltration cartridge (Millipore, Merck, Billerica, MA, USA).

### 4.2. Protocol for Preparation of ε-Polycaprolactone and Collagen Type I Conjugation

The conjugation protocol, shown in Figure 1, was optimised following the steps of a previous work by Wiens et al. [22]. Briefly, 2% w/v PCL was dissolved in 50:50 v/v DMF/DCM (2 g of PCL in 100 mL of DCM/DMF solution) for 2 h. Then, 0.19 g of DCC and 0.053 g of NHS were dissolved in two separate bottles in (50:50 v/v) DMF/DCM in order to obtain two 0.5 M solutions. An amount of 2.5 mM of NHS and 2.5 mM of DCC (previously dissolved in 50:50 v/v DMF/DCM) were added to the PCL solution and then gently stirred for 12 h. Then, the unreacted DCC/NHS residual and the insoluble dicyclohexylurea (DCU) by-product were removed by dialysis against ~10 volumes of DMF/DCM (50:50 v/v) with a cellulose dialysis tube (12–14 kDa), gently stirred for 24 h. Type I collagen (0.5% w/v) was dissolved in 3% v/v acetic acid and stirred for 2 h at room temperature. Collagen solution was then added dropwise to the activated PCL solution and pH adjusted to collagen’s isoelectric point (7.5–7.7) with 3 M NaOH. The resulting gummy-like material (PCL-*g*-Coll) was washed several times in ethanol (30%, v/v) and isopropanol (30%, v/v) and, finally, vacuum dried under a chemical hood.

### 4.3. Preparation of Dense Films by Solvent Casting

An amount of 2% w/v PCL-*g*-Coll synthesised material was dissolved in chloroform for 30 min, then poured into a glass petri dish and left overnight under a chemical hood for solvent evaporation. The same amount of pure PCL was dissolved in chloroform and left to dry in preparation for use as control film. A further control blend film (PCL/Coll) was prepared by dissolving 2% w/v PCL in chloroform with the addition of 0.5% w/v collagen. Then, the solution was titrated in order to achieve a final pH of 7.5–7.7 with the addition of 3 M NaOH drops before pouring into a Petri dish. The blend film was washed several times in ethanol (30%, v/v) and isopropanol (30%, v/v) (as reported above for the conjugated material) in order to simulate the same conditions. All the obtained cast films were stored at 5 °C before further investigation.

### 4.4. Preparation of Dense Films by Spin Coating

For biological tests, thin and homogeneous films were prepared by spin coating (CSS-05, PI-Kem Ltd., Tamworth, UK) on circular microscope glass coverslips (10 mm diameter). PCL, PCL-*g*-Coll and PCL/Coll solutions (2% w/v) were prepared after dissolution in chloroform. Then, 60 μL of each solution was deposited onto the glass substrate and spin-coated at 1000 rpm for 3 s followed by 10 s at 7000 rpm. The procedure was repeated twice.

### 4.5. Electrospun Membranes

The electrospun membranes were initially spun as previously described [4,5]. Briefly, PCL and PCL-*g*-Coll were dissolved in chloroform (Fisher Scientific, Loughborough, UK) by gentle stirring at room temperature to produce a solution of suitable viscosity for electrospinning at a concentration of 10% (w/v). Polymer solutions were loaded into 5 mL plastic syringes (Becton Dickinson, Oxford, UK) and delivered at a constant feed rate of 2500 µL/h, using a programmable syringe pump (Spraybase, Dublin, Ireland) via PTFE tubing (0.8 mm) to a blunt tipped stainless steel needle with an internal diameter of 0.9 mm (20G) (Spraybase, Dublin, Ireland). The needle was in turn connected to a positive high voltage unit (Spraybase, Dublin, Ireland) and solutions were electrospun with an applied voltage between 10 and 15 kV using a voltage generator (Spraybase, Dublin, Ireland). Fibres were deposited onto a grounded fixed collector at a distance of 180 mm from the tip of the needle, coated in 50 µm thick aluminium foil. Electrospinning was performed at an air temperature of 25 °C and a relative humidity of 25%. This resulted in a mat of membrane that was between 60 and 80 µm thick. Membranes were subsequently dried under vacuum at room temperature for 48 h to ensure any residual solvent was completely removed. In this study, all membranes were produced within a tissue engineering laboratory at the University of Bradford, UK.

### 4.6. Physical–Chemical Characterization

#### 4.6.1. Fourier Transform Infrared Spectroscopy in Attenuated Total Reflection Mode (ATR-FTIR)

ATR-FTIR analysis was performed on a Spectrum Two PE instrument using the Universal ATR accessory (Single Reflection Diamond) (PerkinElmer Inc., Waltham, MA, USA) in a range of 4000 to 600 cm^−1^ (resolution 4 cm^−1^). Dried samples were analysed without any preliminary preparative step.

#### 4.6.2. X-ray Photoelectron Spectroscopy (XPS)

XPS spectra were acquired on Theta Probe (Thermo Scientific, Loughborough, UK), equipped with a microfocused AlKa X-ray source (1486.6 eV), and operated with a 400 µm spot size (100 W power). Process parameters were 200 eV pass energy, 1 eV step size and 50 ms dwell time in not angle-resolved lens mode. At least three single areas were evaluated on each membrane surface. Moreover, high resolution spectra were acquired with 40 eV pass energy, 0.1 eV step size and 200 ms dwell time.

#### 4.6.3. Contact Angle Measurement

Contact angle analysis in static conditions was carried out using a CAM 200 KSV instrument (KSV Instruments, Helsinki, Finland), using Drop Shape Analysis System DSA 10 software (Version V2.0-02, KRUSS GmbH, Hamburg, Germany). For all analyses, distillate water drops (3 μL) were used. At least three measurements for each sample (in triplicate) were averaged. 

#### 4.6.4. Sirius Red Method

The presence of collagen was qualitatively identified by using the Sirius red-based colorimetric method. Samples of PCL, PCL/Coll blend and PCL-*g*-Coll were incubated into 1 mL of Sirius red dye reagent contained in a cap tube for 30 min, in order to form a collagen–dye complex following company protocol (Sircol™, Biocolor Ltd., Carrickfergus, UK). The red dye solution was drained and the stained samples were washed three times with ice-cold washing solution in order to remove the unbound dye. Then, the washing solution was removed and samples were placed onto microscope glass slides. Samples were imaged onto an optic microscope DMLB-Leica (Meyer Instruments, Houston, TX, USA) bright field light Microscope combined with Camera-Advanced SPOT.

#### 4.6.5. Tensile Tests

Tensile tests were performed on the fabricated films, previously cut into rectangular shapes (10 mm × 50 mm). The thickness of the films was measured with a micrometer with a precision of 0.01 mm. A tensile test was conducted on an Instron 858 test system (Eden Prairie, MN, USA) equipped with a 1 kN load cell under a cross-head speed of 10 mm/min. The gauge length of the samples was set at 40 mm. Tensile stress (σ) was calculated based on the apparent cross-sectional area of the membranes. Six samples were tested for each type of membrane. The following mechanical properties were recorded or calculated from the stress–strain (σ–ε) curves in which the tensile modulus was calculated from the slope of the initial linear part of the curve.

#### 4.6.6. In Vitro Biodegradation Studies

The in vitro degradation tests were performed in Phosphate Buffered Saline (PBS) solution at 37 °C. Films’ weight losses during degradation were measured by the changes in dry weight after incubation for specified time periods. The films were removed, rinsed in bi-distilled water and dried under a hood for 48 h and weighted using an analytical balance (Kern ABT 220, Kern & Sohn, Stuttgart, Germany). All the experiments were done in triplicate on samples cut into squares of 1 cm^2^.

#### 4.6.7. Morphological Analysis by Scanning Electron Microscopy

The morphological analysis of the samples was performed by Scanning electron microscopy (SEM, HITACHI TM3030, Maidenhead, UK). The diameters of the electrospun fibres were evaluated on at least five SEM micrographs using ImageJ software (Version 1.51k).

### 4.7. Biological Evaluation

#### 4.7.1. Cell Culture

L929 fibroblasts were grown at 37 °C, 5% CO_2_, in Dulbecco’s Modified Eagle Medium (DMEM, Sigma, Gillingham, UK) supplemented with 10% foetal bovine serum (FBS), 2 mM l-glutamine and 1% antibiotic mixture containing penicillin and streptomycin (100 μ/mL). In order to perform biocompatibility assays, spin-coated films on a glass coverslip were prepared as described previously, positioned in 24-well plates and UV-sterilised (Plasma Cleaner PDC-32 g) for 15 min on each side. A suspension of 4 × 10^4^ cells in DMEM was seeded dropwise on the top surface of the samples and incubated at 37 °C, 5% CO_2_ for 30 min. Then, fresh DMEM was added up to 500 µL volume.

#### 4.7.2. Cytocompatibility Assays

Cell viability was assessed with the live/dead staining (LIVE/DEAD^®^ Cell Imaging Kit, Life Technologies, Thermo Fisher Scientific, Waltham, MA, USA) at days 1, 3 and 7. According to the manufacturer’s protocol, samples were washed with phosphate buffered saline (PBS, Sigma-Aldrich, Gillingham, UK) and stained with 150 µL solution of 4 µM Ethidium homodimer-1 and 2 µM calcein in PBS. After 35 min of incubation at room temperature, cells were imaged with a Leica DMLB fluorescence microscope using FITC and Rhodamine filters to detect calcein (ex/em 488 nm/515 nm) and Ethidium homodimer-1 (ex/em 570 nm/602 nm), respectively.

Cell viability was evaluated by using the MTT assay (Sigma Aldrich, Gillingham, UK). For this purpose, the culture media were removed and 200 μL of 1 mg/mL MTT in DMEM (phenol red and serum free) was added to each well. Samples treated with MTT reagent were incubated for 4 h at 37 °C. Then, media were carefully removed and 100 μL of isopropanol was added to each well to solubilise the tetrazolium crystals. The multi-well plate was covered with tinfoil and agitated on an orbital shaker for 30 min. Solubilised formazan (100 μL) was transferred to a 96-multiwell plate and the light absorbance read at λ = 560 nm using a Sunrise Elisa plate reader (XFLUOR4 V4.51, Tecan Life Sciences Mannedorf, Switzerland). The absorbance values were normalised using tissue culture plastic as control.

#### 4.7.3. Cytoskeletal Staining

Culture media were removed from each well and samples were washed twice with Dulbecco’s Phosphate Buffered Saline (DPBS) (Lonza, Slough, UK). Cells that adhered to the sample surface were fixed in 4% (w/v) paraformaldehyde (Sigma-Aldrich, Gillingham, UK) in PBS preheated at 37 °C for 20 min. Subsequently, fixative solution was drained and samples were washed three times with DPBS/0.1% (v/v) Tween 20. Samples were blocked in 3% (v/v) goat serum (Sigma Aldrich, Gillingham, UK) in DPBS/0.1% Tween 20 for 30 min. The vinculin primary antibody (ABfinity™ Rabbit Monoclonal, Cat. 700062, Thermo Fisher, Loughborough, UK) was added (300 μL) and incubated for 120 min at room temperature, and then washed three times with DPBS/0.1% (v/v) Tween 20. Vinculin secondary antibody (Goat anti-Rabbit IgG (H + L), Secondary Antibody, FITC conjugate F-2765, Thermo Fisher) was then added (200 μL) and incubated for 30 min at room temperature, followed by a washing step with DPBS/0.1% (v/v). Samples were incubated with 200 µL of Rhodamine phalloidin (Sigma Aldrich, Gillingham, UK) in DPBS/0.1% Tween 20 (1:1000) for 20 min. Samples were then washed three times with DPBS/0.1% (v/v) Tween 20 and once with ultrapure water. Cell nuclei were visualised with DAPI (Sigma Aldrich) included in the anti-fade mounting medium (Vector Labs. Inc., Peterborough, UK). Samples were mounted onto microscope glass slides (VWR, East Grinstead, UK) and imaged on a fluorescent microscope DMLB-Leica Fluorescent light Microscope (Meyer Instruments, Houston, TX, USA) combined with Camera-Advanced SPOT (Meyer Instruments, Houston, TX, USA).

### 4.8. Statistical Analysis

Tests were performed at least three times for each membrane. All data were expressed as mean ± standard deviation (SD). Statistical analysis was determined by using Graph pad Prism 6 software (7.01 GraphPad Software, Inc., La Jolla, CA, USA). The statistical differences between groups were calculated using Kruskal–Wallis One-Way ANOVA analysis of variance on ranks test. Statistical significance was declared at * *p* < 0.05 and ** *p* < 0.001.

## Figures and Tables

**Table 1:** Atomic concentration (%) of the core-levels of PCL, blend and conjugated samples.

Sample	288.9 eV C=O (%)	288.4 eV N–C=O (%)	286.5 eV C–O (%)	286.1 eV C–N (%)	284.9 eV C–H (%)
PCL	10.17 ± 0.13	-	16.52 ± 0.42	–	73.31 ± 0.41
PCL/Coll	4.93 ± 0.42	3.59 ± 0.87	9.69 ± 1.65	1.55 ± 0.19	76.91 ± 1.73
PCL-*g*-Coll	2.86 ± 0.81	8.52 ± 0.63	7.64 ± 1.78	8.96 ± 2.02	71.82 ± 1.27

## References

[1] Dhandayuthapani B., Yoshida Y., Maekawa T., Sakthi Kumar D. (2011). Polymeric scaffolds in tissue engineering application: A review. Int. J. Polym. Sci..

[2] Pham Q.P., Sharma U., Mikos A.G. (2006). Electrospinning of polymeric nanofibers for tissue engineering applications: A review. Tissue Eng..

[3] Ortega I., Sefat F., Deshpande P., Paterson T., Ramachandran C., Ryan A.J., MacNeil S., Claeyssens F. (2014). Combination of microstereolithography and electrospinning to produce membranes equipped with niches for corneal regeneration. J. Vis. Exp..

[4] Sefat F., McKean R., Deshpande P., Ramachandran C., Hill C.J., Sangwan V.S., Ryan A.J., MacNeil S. (2013). Production, sterilisation and storage of biodegradable electrospun PLGA membranes for delivery of limbal stem cells to the cornea. Procedia Eng..

[5] Deshpande P., Ramachandran C., Sefat F., Mariappan I., Johnson C., McKean R., Hannah M., Sangwan V.S., Claeyssens F., Ryan A.J. (2013). Simplifying corneal surface regeneration using a biodegradable synthetic membrane and limbal tissue explants. Biomaterials.

[6] Gentile P., Ferreira A.M., Callaghan J.T., Miller C.A., Atkinson J., Freeman C., Hatton P.V. (2017). Multilayer nanoscale encapsulation of biofunctional peptides to enhance bone tissue regeneration in vivo. Adv. Healthc. Mater..

[7] Zhang Y.Z., Venugopal J., Huang Z.M., Lim C.T., Ramakrishna S. (2005). Characterization of the surface biocompatibility of the electrospun PCL-collagen nanofibers using fibroblasts. Biomacromolecules.

[8] Ferreira A.M., Gentile P., Toumpaniari S., Ciardelli G., Birch M.A. (2016). Impact of collagen/heparin multilayers for regulating bone cellular functions. ACS Appl. Mater. Interfaces.

[9] Abedalwafa M., Wang F., Wang L., Li C. (2013). Biodegradable poly-epsilon-caprolactone (PCL) for tissue engineering applications: A review. Rev. Adv. Mater. Sci..

[10] Cipitria A., Skelton A., Dargaville T.R., Dalton P.D., Hutmacher D.W. (2011). Design, fabrication and characterization of PCL electrospun scaffolds—A review. J. Mater. Chem..

[11] Ji W., Yang F., Seyednejad H., Chen Z., Hennink W.E., Anderson J.M., van den Beucken J.J.J.P., Jansen J.A. (2012). Biocompatibility and degradation characteristics of PLGA-based electrospun nanofibrous scaffolds with nanoapatite incorporation. Biomaterials.

[12] Ferreira A.M., Gentile P., Chiono V., Ciardelli G. (2012). Collagen for bone tissue regeneration. Acta Biomater..

[13] Dai N.T., Williamson M.R., Khammo N., Adams E.F., Coombes A.G.A. (2004). Composite cell support membranes based on collagen and polycaprolactone for tissue engineering of skin. Biomaterials.

[14] Mahjour S.B., Sefat F., Polunin Y., Wang L., Wang H. (2016). Improved cell infiltration of electrospun nanofiber mats for layered tissue constructs. J. Biomed. Mater. Res. Part A.

[15] Dippold D., Cai A., Hardt M., Boccaccini A.R., Horch R., Beier J.P., Schubert D.W. (2017). Novel approach towards aligned PCL-collagen nanofibrous constructs from a benign solvent system. Mater. Sci. Eng. C.

[16] Lee S.J., Liu J., Oh S.H., Soker S., Atala A., Yoo J.J. (2008). Development of a composite vascular scaffolding system that withstands physiological vascular conditions. Biomaterials.

[17] Tillman B.W., Yazdani S.K., Lee S.J., Geary R.L., Atala A., Yoo J.J. (2009). The In Vivo stability of electrospun polycaprolactone-collagen scaffolds in vascular reconstruction. Biomaterials.

[18] Çapkın M., Çakmak S., Kurt F.Ö., Gümüşderelioğlu M., Şen B.H., Türk B.T., Deliloğlu-Gürhan S.İ. (2012). Random/aligned electrospun PCL/PCL-collagen nanofibrous membranes: Comparison of neural differentiation of rat AdMSCs and BMSCs. Biomed. Mater..

[19] Chong E.J., Phan T.T., Lim I.J., Zhang Y.Z., Bay B.H., Ramakrishna S., Lim C.T. (2007). Evaluation of electrospun PCL/gelatin nanofibrous scaffold for wound healing and layered dermal reconstitution. Acta Biomater..

[20] Kim M., Kim G.H. (2015). Electrohydrodynamic direct printing of PCL/collagen fibrous scaffolds with a core/shell structure for tissue engineering applications. Chem. Eng. J..

[21] Cai Y., Li J., Poh C.K., Tan H.C., San Thian E., Fuh J.Y.H., Sun J., Tay B.Y., Wang W. (2013). Collagen grafted 3D polycaprolactone scaffolds for enhanced cartilage regeneration. J. Mater. Chem. B.

[22] Wiens M., Elkhooly T.A., Schröder H.-C., Mohamed T.H.A., Müller W.E.G. (2014). Characterization and osteogenic activity of a silicatein/biosilica-coated chitosan-graft-polycaprolactone. Acta Biomater..

[23] Ferreira A.M., Gentile P., Sartori S., Pagliano C., Cabrele C., Chiono V., Ciardelli G. (2012). Biomimetic soluble collagen purified from bones. Biotechnol. J..

[24] Sionkowska A., Kozłowska J. (2010). Characterization of collagen/hydroxyapatite composite sponges as a potential bone substitute. Int. J. Biol. Macromol..

[25] Wen F., Chang S., Toh Y.C., Teoh S.H., Yu H. (2007). Development of poly(lactic-co-glycolic acid)-collagen scaffolds for tissue engineering. Mater. Sci. Eng. C.

[26] Ryu S.R., Noda I., Jung Y.M. (2011). Positional fluctuation of IR absorption peaks: Frequency shift of a single band or relative intensity changes of overlapped bands. Am. Lab..

[27] Sionkowska A. (2011). Current research on the blends of natural and synthetic polymers as new biomaterials: Review. Prog. Polym. Sci..

[28] Sabir M.I., Xu X., Li L. (2009). A review on biodegradable polymeric materials for bone tissue engineering applications. J. Mater. Sci..

[29] Lutolf M.P., Hubbell J.A. (2005). Synthetic biomaterials as instructive extracellular microenvironments for morphogenesis in tissue engineering. Nat. Biotechnol..

[30] Nomura S., Hiltner A., Lando J.B., Baer E. (1977). Interaction of water with native collagen. Biopolymers.

[31] Noris-Suárez K., Lira-Olivares J., Ferreira A.M., Feijoo J.L., Suárez N., Hernández M.C., Barrios E. (2007). In vitro deposition of hydroxyapatite on cortical bone collagen stimulated by deformation-induced piezoelectricity. Biomacromolecules.

[32] Gul-E-Noor F., Singh C., Papaioannou A., Sinha N., Boutis G.S. (2015). The behavior of water in collagen and hydroxyapatite sites of cortical bone: Fracture, mechanical wear, and load bearing studies. J. Phys. Chem. C.

[33] Nyman J.S., Roy A., Shen X., Acuna R.L., Tyler J.H., Wang X. (2006). The influence of water removal on the strength and toughness of cortical bone. J. Biomech..

[34] Even-Ram S., Yamada K.M. (2005). Cell migration in 3D matrix. Curr. Opin. Cell Biol..

[35] Cao D., Wu Y.-P., Fu Z.-F., Tian Y., Li C.-J., Gao C.-Y., Chen Z.-L., Feng X.-Z. (2011). Cell adhesive and growth behavior on electrospun nanofibrous scaffolds by designed multifunctional composites. Coll. Surf. B.

[36] Sousa I., Mendes A., Pereira R.F., Bártolo P.J. (2014). Collagen surface modified poly(*ε*-caprolactone) scaffolds with improved hydrophilicity and cell adhesion properties. Mater. Lett..

[37] Lee J.-J., Yu H.-S., Hong S.-J., Jeong I., Jang J.-H., Kim H.-W. (2009). Nanofibrous membrane of collagen–polycaprolactone for cell growth and tissue regeneration. J. Mater. Sci..

[38] Deitzel J.M., Kleinmeyer J., Harris D.E.A., Tan N.C.B. (2001). The effect of processing variables on the morphology of electrospun nanofibers and textiles. Polymer.

[39] Duan H., Feng B., Guo X., Wang J., Zhao L., Zhou G., Liu W., Cao Y., Zhang W.J. (2013). Engineering of epidermis skin grafts using electrospun nanofibrous gelatin/polycaprolactone membranes. Int. J. Nanomed..

